# *Escherichia coli* Antimicrobial Susceptibility Reduction amongst HIV-Infected Individuals at the University Teaching Hospital, Lusaka, Zambia

**DOI:** 10.3390/ijerph17103355

**Published:** 2020-05-12

**Authors:** Freeman Chabala, Mutinta Madubasi, Mable Mwale Mutengo, Njeleka Banda, Kaunda Yamba, Patrick Kaonga

**Affiliations:** 1 The Institute of Basic and Biomedical Sciences, Levy Mwanawasa Medical University, Lusaka 10101, Zambia; mmutengo@yahoo.com; 2 Department of Applied Sciences, Lusaka Apex Medical University, Lusaka 10101, Zambia; tintamadubansi9@gmail.com; 3 Department of Pathology and Microbiology, School of Medicine, University of Teaching Hospital, Lusaka 10101, Zambia; njelekabanda@yahoo.co.uk (N.B.); kaundayamba@gmail.com (K.Y.); 4 Tropical Gastroenterology and Nutrition Group, University Teaching Hospital, Lusaka 10101, Zambia; patrickkaonga@gmail.com; 5 Department of Internal Medicine, School of Medicine, University of Zambia, Lusaka 10101, Zambia

**Keywords:** *Escherichia coli*, antimicrobial susceptibility, Human Immunodeficiency Virus, bacterial gastroenteritis, Zambia

## Abstract

Increased antimicrobial resistance among Human Immunodeficiency Virus (HIV)-infected individuals to commonly used antibiotics in the treatment of gastroenteritis is a public health concern, especially in resource-limited settings. We set out to compare the antimicrobial susceptibility pattern of *Escherichia coli (E. coli)* isolates from HIV-infected and HIV-uninfected individuals at a tertiary hospital in Lusaka, Zambia. An analytical cross-sectional study was conducted at the University Teaching Hospital from May 2019 to August 2019. Stool samples were screened, and 79 HIV-infected individuals matched by age and sex with 84 HIV-uninfected individuals that presented with *E. coli* associated gastroenteritis were studied. Demographics were collected from the Laboratory Information System (LIS) and stool samples were collected in a sterile leak-proof container. Samples were cultured and only those where *E. coli* was isolated were included in the study and tested for antimicrobial susceptibility by the Kirby–Bauer disk diffusion technique. HIV-positive individuals were 3 times (adjusted odds ratio (AOR) = 3.17; 95% CI (1.51, 6.66); *p* < 0.001) more likely to be resistant to quinolones compared with their HIV-negative counterparts. Similarly, HIV-positive individuals were almost 4 times (AOR = 3.97, 95% CI (1.37, 11.46); *p* = 0.011) more likely to have multidrug-resistant *E. coli* compared with those who were HIV-negative. HIV infection was associated with reduced *E. coli* susceptibility to commonly used antibiotics, and most cases showed resistance.

## 1. Introduction

Infection with the Human Immunodeficiency Virus (HIV) is a public health problem that is associated with immunosuppression and an augmented predisposition to opportunistic infections [1,2]. *Escherichia coli* (*E. coli*) is one of the most common etiological agents of bacterial gastroenteritis (GE) amongst HIV-infected individuals, whose disease presents with varying severity [3]. Approximately 50% of HIV-infected individuals succumb to bacterial GE, whose disease severity depends on many factors and has high attributable mortality, especially in resource-poor settings [4,5]. On one hand, Highly Active Antiretroviral Therapy(HAART), a group of different classes of drugs that are used in the treatment of HIV [6], and prophylactic management of opportunistic infections have significantly improved clinical outcomes and overall survival of HIV-infected individuals; others have reported reduced diarrhoeal episodes in bacterial GE [7]. On the other hand, the treatment of diarrhoea in HIV-infected individuals remains a major challenge in this era of marked antimicrobial resistance [8,9]. This problem is ascribed to frequent visits to health facilities for medical check-ups and continuous exposure to antibiotic therapy and prophylaxis [9,10].

A Zambian study conducted during the pre-HAART era reported that Enterobacteriaceae including nontyphoidal Salmonellae, *Shigella flexineri,* and *Shigella dysenteriae* organisms had reduced antimicrobial susceptibility while *E. coli* did not [11], but studies elsewhere have shown increased antimicrobial resistance in *E. coli* [10,12]. However, after 17 years it was unclear whether the situation had changed or not; therefore, we set out to determine the antibiotic susceptibility pattern of *E. coli* isolated among HIV-infected individuals attending a tertiary hospital in Lusaka, Zambia. Findings would provide baseline data to inform a local antibiogram and provide updated specific antibiotics that are currently sensitive to *E. coli* among HIV-infected individuals.

## 2. Materials and Methods

### 2.1. Study Setting

This was a hospital-based analytical cross-sectional study conducted at the University Teaching Hospital (UTH), Lusaka, Zambia, from May 2019 to August 2019. The UTH is the largest referral hospital in Zambia [13]. About 68,000 individuals aged between 15 and 19 years are living with HIV and, according to the Zambia Population-Based HIV Impact Assessment (ZAMPHIA), the prevalence of the disease is 14.9% in this population [14].

### 2.2. Study Population

We screened and consecutively enrolled HIV-positive and HIV-negative individuals that presented with GE due to *E. coli.* We matched the cases to the controls by age and sex.

### 2.3. Sample Size Justification

**Figure 1:**
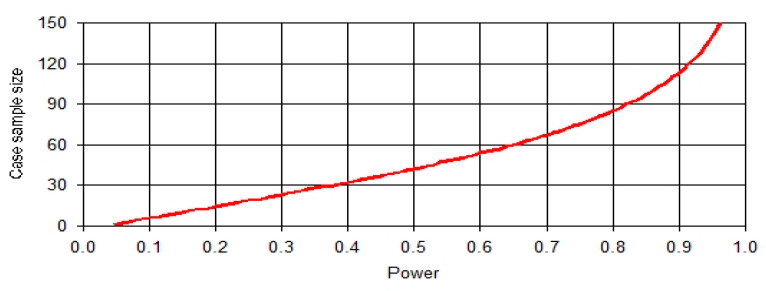
Graph of sample size against power

Sample size calculation was done using the PS Power and Sample Size Calculations (Version 3.0 William D. Dupont and Walton D. Plummer). We designed an analytical cross-sectional study of HIV-positive (cases) and HIV-negative (controls) with one control per case. Prior research findings indicated that the prevalence of multidrug resistance amongst HIV-negative patients was 0.083 [15]. Assuming that the true prevalence rate of multidrug resistance in HIV-positive patients was 0.24, a sample size of 85 HIV-positive patients and 85 HIV-negative patients was required to reject the null hypothesis that these two proportions are equal with 0.8 power (Figure 1.) and 0.05 Type I error probability. We used an uncorrected chi-squared statistic to evaluate this null hypothesis using the formula below.

(1) $$2N=\frac{2{\left\{Z_{\alpha /2}\sqrt{2\bar{P}\left(1-\bar{P}\right)}+Z_{\beta }\sqrt{P_{A}\left(1-P_{A}\right)+P_{B}\left(1-P_{B}\right)}\right\}}^{2}}{{\left(P_{B}-P_{A}\right)}^{2}}$$

(2) $$\bar{P}=\frac{\left(P_{A}-P_{B}\right)}{2}$$

*P_A_* = proportion of multidrug resistance among HIV-negative patients

*P_B_* = proportion of multidrug resistance among HIV-positive patients

We want to test H_0_: PA = PB vs. H_1_: *P_A_* ≠ *P_B_* (P = true value)

With significance level Z_α/2_ and power = 1 − β to detect a difference of δ = *P_A_* − *P_B_*

### 2.4. Sampling Strategy

We made an excel spreadsheet of HIV-positive and HIV-negative patients infected with *E. coli* in the period between January 2019 and March 2019. We had a total of 186 patients infected with *E. coli* but only 163 had known HIV status; 84 HIV-positive and 79 HIV-negative. We included all the participants in the study with known HIV status and excluded the 23 with unknown HIV status.

### 2.5. Data and Specimen Collection

Demographic characteristics such as age, sex, HIV status, and severity of diarrhoea were obtained from the Laboratory Information System (LIS) model Build 981 (Disa*Lab, Cape Town, South Africa). A single stool specimen was obtained from each participant in a sterile leak-proof, disinfectant-free container and transported within 2 hours of collection to the laboratory for analysis and culture.

### 2.6. Isolation and Identification of E. coli

*E. coli* isolation and phenotypic characterization were conducted using the recommended culture method and all the biochemical tests were performed as described elsewhere [16]. Stool samples were cultured on Xylose Lysine Deoxycholate (XLD) and MacConkey (MAC) and Xylose following enrichment on Selenite-F Broth (Thermo Scientific., Hampshire, United Kingdom). *E. coli* was characterized using biochemical tests and serotyping was performed using commercial anti-sera (BD Diagnostics, Franklin Lakes, New Jersey, USA).

### 2.7. Antimicrobial Susceptibility Test

Antimicrobial susceptibility was tested using the Kirby–Bauer disc diffusion method as recommended by the Clinical and Laboratory Standards Institute (CLSI) [17,18]. In summary, one to three *E. coli* colonies were inoculated into a nutrient broth to prepare inoculums; the broths were incubated at 37 °C for 24 h. Turbidity of broths was standardized at 0.5 McFarland using sterile phosphate-buffered saline (pH 7.2). The standardized suspension was inoculated on Mueller–Hinton agar plates and incubated for 24 h at 37 °C. Antibiotic discs representing commonly prescribed antimicrobials at the study site included ciprofloxacin (5 μg), piperacillin/tazobactam (30 μg), trimethoprim-sulfamethoxazole (1.25/23.75 μg), nalidixic acid (30 μg), amikacin (30 μg), ampicillin (30 μg), tobramycin (30 μg), and ceftriaxone (30 μg) (Thermo Scientific, Bedford, MA, USA). The diameter of the inhibition zone was measured using a calliper and interpreted according to the standard [18]. A reference strain of *E. coli* (ATCC-25922) was used as a control. Some studies define multidrug-resistant *E.coli* as having resistance to three families of drugs, while others define it as having resistance to cotrimoxazole, that is, having extended-spectrum beta-lactamase (ESBL) activity and resistance to third- and fourth-generation cephalosporins and fluoroquinolones with or without carbapenem resistance [15,19]. In this study, the *E.coli* isolates were predominantly resistant to cotrimoxazole; therefore, we defined multidrug resistance for our analysis as resistance to aminoglycosides and fluoroquinolones, which are amongst the main drugs of choice in the treatment of infection due to *E. coli* in this setting.

### 2.8. Statistical Analysis

Data were entered in Epi Data version 3.1 (Epi Data Corp., Odense, Denmark) and exported into Stata 15 (StataCorp., College Station, Texas, USA) for analysis. The bootstrap median and a 95% confidence interval were used to estimate the central tendency of continuous variables, such as age, while frequencies and percentages were used to summarize categorical variables. The Shapiro–Wilk test was used to test for normality for continuous variables. An unpaired T-test was used for comparing two normally distributed independent variables and the Wilcoxon–Mann–Whitney rank-sum test was used in cases where the dependent variable was not normally distributed. We used multiple logistic regression to estimate the odds of having resistance to aminoglycosides, resistance to quinolones, and resistance to both aminoglycosides and quinolones on condition for age, sex, HIV status, and severity of diarrhoea in the multiple regression models.

### 2.9. Ethical Approval

Permission to conduct the study was sought from the UTH management and ethical approval was obtained from ERES Converge (Ref no. 2018-Nov-042).

## 3. Results

There were 84 HIV-positive and 79 HIV-negative individuals. HIV-positive individuals were younger than HIV-negative individuals by a median age of 2 years *p* < 0.001: the median age of HIV-positive individuals was 13 years (bootstrap 95% CI (12.5, 13.5) vs. HIV-negative 16 years (bootstrap 95% CI (15.1, 16.9). In the HIV-positive group, 46 (54.7%) were females while in the HIV-negative group 41 (51.9%) were. The majority of individuals had acute diarrhoea in both groups: 38 (49.4%) and 49 (58.3%) among HIV-positive and HIV-negative individuals, respectively (Table 1).

### 3.1. Simple Logistic Regression Models

Upon the comparison of the antimicrobial susceptibility of *E. coli* between HIV-positive and HIV-negative individuals, findings demonstrated marked antimicrobial resistance among HIV-positive individuals following simple logistic regression analyses. *E. coli* isolates from HIV-positive individuals were 1.9 times more likely to be resistant to Amikacin compared with those from HIV-negative individuals (95% CI [1, 3.5], *p* = 0.05). Isolates from HIV-positive individuals were 2.6 times more likely to be resistant to Piperacillin/Tazobactam compared with those from HIV-negative individuals (95% CI [1.4, 5], *p* = 0.003). Isolates from HIV-positive individuals were 4.9 times more likely to be resistant to Cotrimoxazole compared with those from HIV-negative individuals (95% CI (2.2, 10.9), *p* < 0.001). Isolates from HIV-positive individuals were 1.8 times more likely to be resistant to Ciprofloxacin compared with those from HIV-negative individuals (95% CI [1, 3.4], *p* < 0.05). Isolates from HIV-positive individuals were 2.5 times more likely to be resistant to Nalidixic acid compared with those from HIV-negative individuals (95% CI [1.3, 4.7], *p* = 0.005). Finally, isolates from HIV-positive individuals were 2.5 times more likely to be resistant to Nalidixic acid compared with those from HIV-negative individuals (95% CI [1.3, 4.7], *p* = 0.005). However, there was no difference in the susceptibility of *E. coli* isolates from HIV-positive and HIV-negative individuals to antibiotics such as Ampicillin, Tobramycin, and Ceftriaxone, *p >* 0.05 (Table 2).

### 3.2. The Multiple Logistic Regression Model

Three multiple logistic regression analyses were done to determine the probability of having resistance to aminoglycosides (amikacin and tobramycin), resistance to quinolones (Nalidixic acid and ciprofloxacin), and resistance to both aminoglycosides and quinolones (multidrug resistance) upon conditioning for age, sex, and clinical diagnosis. Findings showed that there was no association between resistance to aminoglycosides and HIV status, *p* = 0.244. However, HIV-positive individuals were 3 times (adjusted odds ratio (AOR) = 3.17; 95% CI (1.51, 6.66); *p* < 0.001) more likely to be resistant to quinolones compared with their HIV-negative counterparts. Similarly, HIV-positive individuals were almost 4 times (AOR = 3.97, 95% CI (1.37, 11.46); *p* = 0.011) more likely to have multidrug-resistant *E. coli* compared with those who were HIV-negative (Table 3).

## 4. Discussion

*E. coli* is a common cause of bacterial GE worldwide and a major public health problem [1,21]. Amongst HIV-positive individuals, *E. coli* is one of the leading causes of morbidity and mortality, especially in developing countries where sanitation and access to clean and safe water is a challenge. Unfortunately, information about the susceptibility pattern of stool isolates of this organism to commonly used antibiotics in the HIV population is scarce [22]. Literature has demonstrated a decline in antimicrobial susceptibility of bacteria to commonly used antibiotics in HIV-positive compared with HIV-negative individuals, but little information exists to explain this finding and the magnitude of the problem [1,23,24].

In this study, we set out to determine an association between HIV infection and decline in susceptibility of *E. coli* to commonly used antibiotics in Zambia. In the study population, HIV-positive individuals were significantly younger than HIV-negative individuals with a median age of 13 years compared with HIV-negative individuals with one of 16 years. This age difference might concur with allegations that the majority of HIV adolescents could be infected by vertical transmission as most adolescents become sexually active between 15 and 19 years of age. In Ethiopia, the median age for the sexual debut was 16.6 years [25]; in South Africa, it was 16 years for females and 15 years for males [26]; and in Malawi, it was 15 to 19 years [27]. Among the HIV-positive individuals, there was a female preponderance observed that could be due to greater biological susceptibility to HIV infection due to a larger mucous area being at risk of exposure to HIV during sexual intercourse [28] and vulnerabilities created by imbalanced social, cultural, and economic status, especially in developing countries [29].

Chronic diarrhoea was more prevalent among HIV-positive compared with HIV-negative individuals, concurring with reports elsewhere [30]. This finding is attributable to reduced immunity following depletion of Cluster of Differentiation (CD) 4 cells in HIV that permits diseases’ progression and vulnerability to chronicity [5,30]. Furthermore, there was a significant decline in zones of inhibition among antibiotics such as amikacin, ampicillin, tobramycin, piperacillin/tazobactam, ciprofloxacin, and nalidixic acid among HIV-positive compared with HIV-negative individuals. This finding is in conformity with reports from Cameroon that *E. coli* in HIV-positive individuals was mostly resistant to antibiotics compared with HIV-negative individuals [3,10,23]. It was suggested that the finding of resistance to common antibiotics in HIV-positive individuals was probably a result of frequent exposure to antibiotics on follow-ups and inappropriate use as well as misuse of antibiotics due to frequent illness [31,32]. However, the real underpinning mechanism of antimicrobial resistance in HIV could not quite be ascertained and remains contentious. It is an important puzzle to solve considering the lack of clarity as to whether this reduction is true for other bacteria and the magnitude of the problem.

Antimicrobial resistance is one of the global public health concerns and the advent of HIV is an aggravating factor. On one hand, our study showed that, regardless of HIV status, *E. coli* isolates were resistant to ampicillin and tobramycin, which are amongst the most frequently empirically prescribed antibiotics via self-medication; they are prescribed as prophylaxis after surgical procedures and resistance is due to inappropriate use and overuse. It was noted that most of the antibiotics that showed sensitivity amongst HIV-negative individuals proved to be either resistant or intermediate resistant in HIV-positive individuals; this problem becomes more detrimental because currently there is no algorithm specifically for HIV-infected individuals and both the drugs of choice and doses remain the same, provided the aetiology remains the same. Our results from adjusted logistic regression indicated that HIV-positive individuals were more likely to be resistant to quinolones and experience multidrug resistance. There are possible implications for empirical treatment; since for some antibiotics there is no difference given the HIV status while for others HIV-positive individuals have higher rates of resistance, one could argue that there is a high proportion of HIV-positive individuals receiving treatment for bacterial GE due to *E. coli* that may not be benefitting much from the treatment. Instead, treatment is likely propagating antimicrobial resistance. Furthermore, this could give rise to the proliferation of multidrug-resistant *E. coli* variants that can multiply persistently with HIV-positive individuals being a reservoir for infection, thereby exacerbating the public health system distress.

These results suggest the need for further investigation into antimicrobial resistance in HIV, for identifying the mechanistic processes underlying it, and quantifying the magnitude of the problem and effects on clinical and public health outcomes. This study may be an epiphany that sets a benchmark or gives preliminary information about prevailing issues in antimicrobial use amongst HIV-infected individuals in Zambia and possibly other developing countries. Ultimately, we highlight the importance of antimicrobial susceptibility testing for bacteria causing diarrhoea and other bacterial diseases among HIV-positive individuals and the danger of assuming the equal efficacy of antibiotics without considering HIV status. Finally, in order for the HIV population to circumvent negative consequences, the study suggests the need for constant monitoring of antibiotic susceptibility patterns in HIV-infected individuals and the possible need for the re-examination of the current antibiotic algorithm.

## 5. Limitations

Our study has several limitations. First, the study was conducted at one tertiary hospital in Lusaka, Zambia. We therefore advise that any attempts to generalize our findings should be cautious. Second, as our study had an analytical cross-sectional design, no causal relationship could be deduced between HIV and antimicrobial resistance. Third, individuals who presented to the hospital with GE symptoms were seen by different clinicians on duty; this could have created bias on who was sent or not sent to the laboratory for a stool culture.

## 6. Conclusions

HIV infection is associated with the reduced susceptibility of faecal *E. coli* to many families of antibiotics and caution should be taken when treating these individuals as the drugs may not work effectively.

## Figures and Tables

**Table 1:** Demographic characteristics of study participants.

Characteristic	HIV-Positive	HIV-Negative	*p*-Value
Age (years) *	13 (12.5, 13.5)	16 (15.1, 16.9)	<0.001
Sex (%) **			
Male Female	38 (45.3) 46 (54.7)	38 (48.1) 41 (51.9)	0.714
Diarrhoea severity (%) **			
Acute diarrhoea	38 (49.4)	49 (58.3)	
Persistent	18 (9.1)	16 (13.1)	0.665
Chronic diarrhoea	21 (41.5)	24 (28.6)	

HIV = Human Immunodeficiency Virus; * median (Bootstrap 95% confidence interval); ** chi-square test used. Acute diarrhoea (<1 week), Persistent (1–2 weeks), Chronic (>2 weeks) [20].

**Table 2:** Simple Logistic Regression Models of Drug Resistance by Human Immunodeficiency Virus (HIV) Status.

Drug	COR	95% CI	*p*-Value
Amikacin			
HIV-negative	Ref.		
HIV-positive	1.9	1, 3.5	0.05
Ampillicin			
HIV-negative	Ref.		
HIV-positive	1.6	0.6, 4.2	0.311
Tobramycin			
HIV-negative	Ref.		
HIV-positive	1.8	0.9, 3.6	0.100
Piperacillin/Tazobactam			
HIV-negative	Ref.		
HIV-positive	2.6	1.4, 5	0.003
Cotrimoxazole			
HIV-negative	Ref.		
HIV-positive	4.9	2.2, 10.9	<0.001
Ciprofloxacin			
HIV-negative	Ref.		
HIV-positive	1.8	1, 3.4	0.050
Nalidixic acid			
HIV-positive	Ref.		
HIV-positive	2.5	1.3, 4.7	0.005
Ceftriaxone			
HIV-negative	Ref.		
HIV-positive	1.2	0.6, 2.2	0.640

COR = crude odds ratio; Ref. = reference category; CI = confidence interval.

**Table 3:** Multiple Logistic Regression Models of resistance to the family of antimicrobials.

Variable	AOR	*p*-Value	95% CI
**Aminoglycosides**			
HIV Status			
Negative	Ref.		
Positive	1.49	0.244	0.76, 2.92
Sex			
Female	Ref.		
Male	1.09	0.805	0.57, 2.08
Age (years)	0.96	0.125	0.90, 1.01
Diarrhoea			
Persistent	Ref.		
Acute	1.74	0.198	0.75, 4.03
Chronic	2.01	0.155	0.77, 5.25
**Quinolones**			
HIV Status			
Negative	Ref.		
Positive	3.17	<0.001	1.51, 6.66
Sex			
Female	Ref.		
Male	1.36	0.406	0.66, 2.78
Age (years)	1.01	0.827	0.95, 1.07
Diarrhoea			
Persistent	Ref.		
Acute	1.35	0.517	0.54, 3.39
Chronic	1.16	0.771	0.42, 3.26
**Multidrug-resistant**			
HIV Status			
Negative	Ref.		
Positive	3.97	0.011	1.37, 11.46
Sex			
Female	Ref.		
Male	1.13	0.795	0.45, 286
Age (years)	0.99	0.908	0.92, 1.08
Diarrhoea			
Persistent	Ref.		
Acute	1.26	0.703	0.38, 4.20
Chronic	1.00	0.997	0.27, 3.79

AOR = adjusted odds ratio; Ref. = reference category; CI = confidence interval; HIV = Human Immunodeficiency Virus; Acute diarrhoea (<1 week), Persistent (1–2 weeks), Chronic (>2 weeks).
